# Psychophysical Evaluation of Visual Functions of Ex-Alcoholic Subjects After Prolonged Abstinence

**DOI:** 10.3389/fnins.2019.00179

**Published:** 2019-03-06

**Authors:** Isabelle Christine Vieira da Silva Martins, Givago da Silva Souza, Alódia Brasil, Anderson Manoel Herculano, Eliza Maria da Costa Brito Lacerda, Anderson Raiol Rodrigues, Alexandre Antonio Marques Rosa, Dora Fix Ventura, Antonio José de Oliveira Castro, Luiz Carlos de Lima Silveira

**Affiliations:** ^1^Instituto de Ciências da Saúde, Faculdade de Nutrição, Universidade Federal do Pará, Belém, Brazil; ^2^Instituto de Ciências Biológicas, Universidade Federal do Pará, Belém, Brazil; ^3^Núcleo de Medicina Tropical, Universidade Federal do Pará, Belém, Brazil; ^4^Biomedical School, Universidade Ceuma, São Luís, Brazil; ^5^Instituto de Ciências de Saúde, Faculdade de Medicina, Universidade Federal do Pará, Belém, Brazil; ^6^Instituto de Psicologia, Universidade de São Paulo, São Paulo, Brazil

**Keywords:** alcoholism, contrast sensitivity, color vision, abstinence period, psychophysics

## Abstract

Chronic alcohol abuse can lead to a brain damages, and the health status of alcoholics even after a long-term alcohol abstinence is a public health concern. The present study investigated the color vision and spatial luminance contrast sensitivity of a group of 17 ex-alcoholics (46.3 ± 6.7 years old) in long-term alcohol abstinence after having been previously under alcohol dependence for many years. We also investigated the association of impaired psychophysical performance in different tests we applied. The mean time of alcohol consumption was 16.9 ± 5.1 years and the mean abstinence period was 12.4 ± 8.5 years. Achromatic vision of all subjects was evaluated using spatial luminance contrast sensitivity function (CSF) test and color vision was evaluated using Mollon–Reffin color discrimination test (MR) and the Farnsworth–Munsell 100 hue arrangement test (FM100). Relative to controls, the spatial luminance contrast sensitivity was lower in 10/17 of the ex-alcoholic subjects. In the color vision tests, 11/16 ex-alcoholic subjects had impaired results compared to controls in the FM100 test and 13/14 subjects had color vision deficits measured in the MR test. Fourteen subjects performed all visual tests, three subjects had impaired results for all tests, seven subjects had impaired results in two tests, three subjects had visual deficit in one test, and one had normal results for all tests. The results showed the existence of functional deficits in achromatic and chromatic vision of subjects with history of chronic alcoholism after long abstinence. Most subjects had altered result in more than one test, especially in the color vision tests. The present investigation suggests that the damage in visual functions produced by abusive alcohol consumption is not reversed after long term alcohol abstinence.

## Introduction

The abusive alcohol consumption results in death of 2.5 million people per year around the world. Approximately 60 different types of pathologies have significant association with alcohol consumption (World Health Organization [WHO], 2000). In the central nervous system alterations involving the thalamus, hypothalamus, cerebellum, frontal lobe, corpus callosum that develop with tissue atrophy and altered regulation of the neurotransmitter functions have been reported (Estruch et al., 1997; Moselhy et al., 2001; Zahr et al., 2011; Zhao et al., 2011). In the visual system, many diseases can be associated with alcoholic behavior, such as cataract, age-related macular degeneration, diabetic retinopathy and glaucoma (Chong et al., 2008; Kanthan et al., 2010; Lee et al., 2010).

Alcohol causes transient and permanent effects on the visual system. The effects seem to be time dependent. Acute alcohol exposure causes transitory effects in the visual system that show partial or total functional recovery in the scale of minutes to hours (Zulauf et al., 1988). Chronic alcohol consumption leads to effects in the visual system with longer duration or even permanent damage (Verriest et al., 1980; Kapitany et al., 1993).

Luminance contrast sensitivity and color vision have been extensively investigated in subjects after acute and chronic alcohol consumption. Previous studies show that alcohol intake induces significant impairment in the alcohol intake impaired the spatial luminance contrast sensitivity. In addition, some reports described luminance contrast sensitivity impairment at a wide range of spatial frequencies (Roquelaure et al., 1995), while other investigations found visual losses at intermediate to high spatial frequencies (Andre et al., 1994; Nicholson et al., 1995; Cavalcanti and Santos, 2008).

The first descriptions of color vision losses associated with alcoholism were made in patients with liver cirrhosis (Cruz-Coke, 1964; Cruz-Coke and Varela, 1965; Fialkow et al., 1966). Selective color vision losses affecting the blue-yellow color contrast mechanisms were reported by several authors (Cruz-Coke and Varela, 1965; Thuline, 1967; Varela et al., 1969; Sassoon et al., 1970; Cruz-Coke, 1972; Adams, 1978; Russell et al., 1980; Verriest et al., 1980; Zrenner et al., 1986), while others have reported a predominant loss of red-green color vision (Sarraux et al., 1966; Sakuma, 1973; Kapitany et al., 1993) or a diffuse color vision loss (Smith and Layden, 1971; Rothstein et al., 1973; Mergler et al., 1988; Castro et al., 2009; Brasil et al., 2015).

Visual tests are designed to evaluate specific visual properties such as hue ordering (for example, Farnsworth Munsell 100 hue test), chromatic discrimination (for example, Cambridge Colour Test), and luminance contrast sensitivity. As the alcohol consumption seems to affect differently the putative contribution of these visual pathways to visual perception (Zrenner et al., 1986; Zhuang et al., 2012). However, it is not clear if the visual losses occurring in a test will also be present in other tests. There is no description regard association of color vision and luminance vision impairments caused by alcohol intake. We investigated color vision and spatial luminance contrast sensitivity of a group of subjects in long-term alcohol abstinence after having suffered from alcohol dependence for many years in the past. We compared visual performance in the different tests to determine if losses are revealed by all tests to find out if they are equally sensitive to the affected mechanisms. We also evaluated the association between visual tests results and the history of alcoholism, smoking and abstinence.

## Materials and Methods

### Subjects

We evaluated 17 subjects, 13 male e 4 female (between 31 and 60 years, 46.3 ± 6.7-years-old), all volunteers and members of the Alcoholics Anonymous, with history of chronic alcoholism. The CAGE test was used to identify alcohol dependence (Ewing, 1984). The study was approved by the Research Ethics Committee (report #28/2003) of the Núcleo de Medicina Tropical, Universidade Federal do Pará, Brazil. All subjects gave written and informed consent for participation in the study.

Exclusion criteria of ex-alcoholic subjects were presence of eye diseases, neurological and systemic pathologies, exposure to neurotoxic chemical substances such as mercury and organic solvents and use of medical drugs which affect the visual system such as chloroquine, hidroxicloroquina, ethambutol, vigabatrin. An inquiry about frequency of alcohol consumption, the symptoms shown in period of alcohol use and in the abstinence period, time of chronic alcoholism, of alcohol abstinence, of smoking and of smoking abstinence was performed. All subjects were evaluated by an ophthalmologist who examined biomicroscopy, fundoscopy, refractometry, and ocular motility. All subjects had normal visual acuity or corrected to 20/20. The subjects were tested monocularly in a dark room.

### Procedures and Equipments

Three visual psychophysical tests were used to evaluate the visual system of the ex-alcoholic subjects. To evaluate luminance vision, we estimated the spatial luminance contrast sensitivity (*n* = 17), and to evaluate color vision, we estimated the color discrimination ellipses using the Mollon–Reffin (MR) test (*n* = 15) and we also quantified the total error in the Farnsworth–Munsell 100 hue arrangement (FM100) test (*n* = 16).

### Spatial Luminance Contrast Sensitivity

For spatial luminance contrast sensitivity test, we used an IBM model Pentium IV 1.7 GHz, with ANNIHILATOR 2 by CREATIVE and color palette of 24 bits/8 bits per gun to program the test in C++ language. The stimulus was displayed in a color monitor (“21,” SONY Multiscan G420 model, spatial resolution of 1024 × 768 pixels, temporal resolution of 75 Hz, Japan). The monitor calibration was performed with a CS-100A chromameter (Konica Minolta, Mahwah, NJ, United States).

The stimulus was composed by achromatic sine-wave stationary gratings, 6.5° x 5° of visual angle, at eleven spatial frequencies (0.2, 0.5, 0.8, 1, 2, 4, 6, 10, 15, 20 and 30 cpd). The mean chromaticity of the stimulus in the CIE 1976 color space was *u^′^* = 0.182, *v^′^* = 0.474, and the mean luminance of the screen was 43.5 cd/m^2^. The test was performed using the method of adjustment to control the Michelson contrast of the stimulus. Initially, the stimulus was shown in low contrast to the tested subject. The experimenter gradually increased the stimulus contrast until the subject detected the stimulus. The stimulus contrast was then decreased until the subject just ceased to detect the stimulus, to again increase the stimulus contrast. The adjustment to the just perceptible contrast (contrast threshold) was done 6 times. The contrast threshold was the average of 6 measurements.

### Mollon–Reffin Test

Mollon–Reffin test (MR) was written using C++ programming language. The software was developed for an IBM POWERStation RISC 6000 (IBM Corporation, New York, NY, United States). The stimuli were generated using *IBM POWER GT4-24bits-3D*. The stimuli were displayed on *IBM 6091 19i*, with spatial resolution of 1280 × 1024 pixels. The calibration was performed with a CS-100A chromameter (Konica Minolta, Mahwah, NJ, United States).

We estimated color discrimination ellipses around 5 central coordinates in the CIE1976 color space as following: Ellipse 1, *u*^′^ = 0.215; *v*^′^ = 0.531; Ellipse 2, *u*^′^ = 0.219; *v*^′^ = 0.481; Ellipse 3, *u*^′^ = 0.225; *v*^′^ = 0.415; Ellipse 4, *u*^′^ = 0.175; *v*^′^ = 0.485; and Ellipse 5, *u*^′^ = 0.278; *v*^′^ = 0.472. The stimuli were composed by mosaic of circles that had different sizes (0.2° and 0.6° of diameter) and luminance (from 12 to 20 cd/m^2^). A target differed of the surrounded field by the chromaticity content. The chromaticity of the field was the central coordinate, while the chromaticity of the target was modulated in one out of eight chromatic axes that radiated from the central coordinate.

The target was C-shaped with outer diameter of 4.4°, inner diameter of 2.2° and gap of 1° visual angle. The subject’s task was to identify the C gap orientation among four alternatives (up, left, down, right) during 1.5 s. A staircase was used to control the distance between chromaticity of the field and of the target. One hit decreased the length of the vector that linked both chromaticities, and the chromaticity of the target came closer to the chromaticity of the field. One wrong response increased the vector length between the field and target chromaticities, and the chromaticity of the target became more far from the field chromaticity. One hit followed by a mistake or one mistake followed by a hit was considered as one reversal of the staircase. The test ended when 12 reversals were completed. The color discrimination threshold was the mean value of the last 6 reversals of the staircase. The color discrimination thresholds were fitted by an ellipse function using the least square method. The diameter of the circle with equivalent area of the ellipse was the indicator of the color discrimination of each subject.

### Farnsworth–Munsell 100 Hue Arrangement Test

For the Farnsworth–Munsell 100 hue arrangement test (FM100), the equipment used was the same used for the spatial luminance contrast sensitivity spatial test. The stimulus was composed by 85 circles varying in hue with same color saturation. The presentation of the stimulus was separated in 3 moments with 21 hues and 1 moment with 22 hues. Each circle had a size of 1° of visual angle and luminance of 42 cd/m^2^.

At the beginning of each test all caps were shown ordered in hue during 1 min. After this period, the circles were randomly mixed up. The tested subject had begun by choosing the circle whose hue that was closest to that of a reference circle. The procedure was repeated for the succeeding choices by always choosing the circle with the hue most like the last one chosen. After the complete ordering of the circles, the number of errors in hue sequence was quantified by the software (Farnsworth, 1957). The test was done 4 times for each stimulus presentation. The result was the averaged total error of the 4 trials.

### Statistics

The data from ex-alcoholic subjects were compared to data (tolerance intervals) from age matched control subjects for each visual test as follows: spatial luminance contrast sensitivity test (control group; *n* = 44, 43.8 ± 9.4 years), FM100 test (control group; *n* = 52; 42.9 ± 8.8 years), MR test (*n* = 33; 46.2 ± 6.6 years). We quantified the number of ex-alcoholic subjects whose thresholds fell outside the control group tolerance interval for each visual test. The comparison of ex-alcoholic group and control group was done using Kruskal–Wallis test for spatial luminance contrast sensitivity test and using an independent samples *t*-test to compare both group results for FM100 test and for MR test. We use a multivariate Fisher linear discriminant analysis to test which group of tests (color vision or contrast sensitivity) better separate the data from controls and alcoholics. For all statistics was used the Biostat 5.0 software and considered α of 5%.

## Results

### History of Chronic Alcoholism, Smoking and Abstinence

Table 1 shows the age, alcoholism period and smoking profile of the subjects used in the current study. The mean age was 46 years old ± 7, the mean number of years of alcoholism was 17 ± 5 years and of abstinence was 12 ± 9 years. The mean time of smoking was 17 ± 7.2 years and smoking abstinence was 12 ± 9 years. Three subjects never smoked, four are still smokers, one did not declare and 9 quit smoking.

**Table 1:** History of chronic alcoholism, smoking and abstinence.

Code	Age range (years)	Duration of alcoholism (years)	Time of alcohol abstinence (years)	Duration of smoking (years)	Time of smoking abstinence (years)	CSF	MR test	FM-100
ACS101112	46–50	22	9	24	0	Yes	Yes	Yes
AVO100403	41–45	14	13	15	0	Yes	Yes	Yes
EJM101220	36–40	20	4	20	4	Yes	Yes	Yes
EWS100303	51–55	20	6	**	**	Yes	Yes	Yes
FMC090703	56–60	10	20	20	18	Yes	Yes	Yes
FMR090707	51–55	8	23	16	25	Yes	Yes	Yes
FPS100305	36–40	12	9	3	22	Yes	Yes	Yes
HNS101019	41–45	21	6	5	6	Yes	Yes	Yes
#ILR110211	36–40	20	3	23	0	Yes	No	Yes
LFS101201	51–55	16	20	23	17	Yes	Yes	Yes
MES100219	46–50	26	13	26	5	Yes	Yes	Yes
MSS100223	46–50	13	36	16	0	Yes	Yes	Yes
OAC101013	46–50	15	15	**	**	Yes	Yes	Yes
#RSC101103	51–55	18	10	10	5	Yes	No	Yes
SSR101110	41–45	21	6	20	2	Yes	Yes	Yes
VMN101223	46–50	22	4	*	*	Yes	Yes	Yes
□VNR090806	36–40	10	15	**	**	No	Yes	No
Mean	46	17	12	17.0	12			
SD	7	5	9	7.2	9			


*SD, standard deviation. ^∗^, Not declared. ^∗∗^, Never used.*

### Spatial Luminance Contrast Sensitivity Function

**FIGURE 1:**
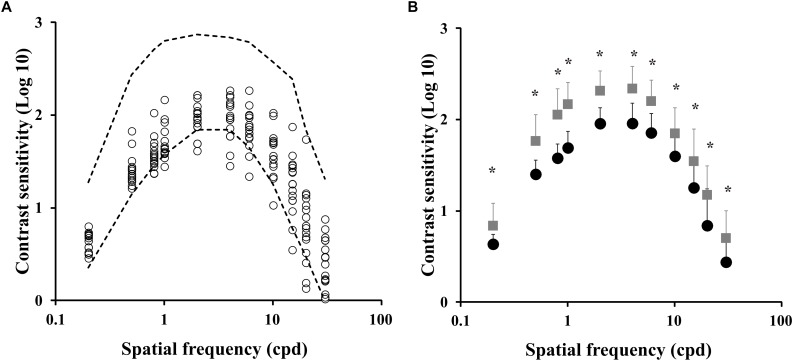
Spatial luminance contrast sensitivity functions of ex-alcoholic subjects. **(A)** Results of the ex-alcoholic subjects (circles) compared to the tolerance interval of the control group (interval between dotted lines). **(B)** Comparison of the mean contrast sensitivity function obtained from control group (gray squares) and ex-alcoholic subjects (black circles). Error bars represent the standard deviation of the mean. ^∗^*p* < 0.05.

Figure 1A shows the spatial luminance contrast sensitivity function estimated from the ex-chronic alcohol consumers (*n* = 17) compared to the tolerance interval of the control group (dotted lines). Figure 1B compares the mean contrast sensitivity function estimated from the control group and from the ex-alcoholics. We observed that the contrast sensitivity of the ex-alcoholics was significantly lower than the control group at 0.5 and 0.8 cpd (*H* = 528.7, *p* < 0.05).

### Farnsworth-Munsell 100 Hue Arrangement Test

**FIGURE 2:**
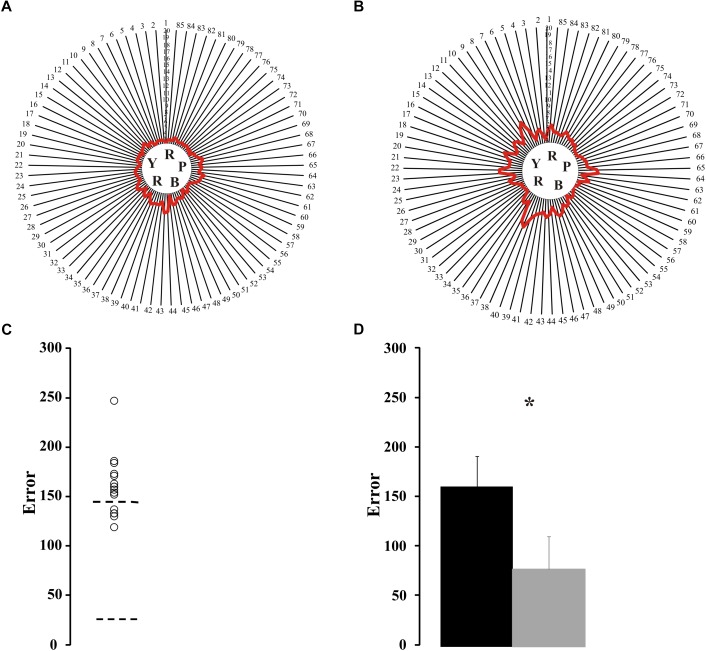
Results of Farnsworth-Munsell 100 hue arrangement test. **(A,B)** Maps of results for the FM100 test from a control subject and an ex-alcoholic. Red lines represent the averaged error for the four trials of each subject. **(C)** Scatter plot for FM100 score estimated from ex-alcoholic subjects (circles). Dotted lines are tolerance interval for the control group. **(D)** Comparison of mean values for the FM100 scores estimated from the control group (gray bar) and ex-alcoholic group (black bar). Error bars represent the standard deviation of the mean. ^∗^*p* < 0.05.

Sixteen ex-alcoholics performed the Farnsworth-Munsell 100 hue arrangement test. Figure 2A,B shows the individual map of errors from a control subject (Figure 2A) and an ex-alcohol consumer (Figure 2B). Figure 2C shows the scattering of the error values of the ex-alcoholics compared to the normative range of the control group (dotted lines). Eleven out of sixteen ex-alcoholics had higher number of errors than the upper tolerance limit of the control group. The mean error of the alcohol consumer group was higher than the mean error of the control group [*t*(66) = 8.85, *p* < 0.05], as shown in the Figure 2D.

### Mollon–Reffin Color Discrimination Test

**FIGURE 3:**
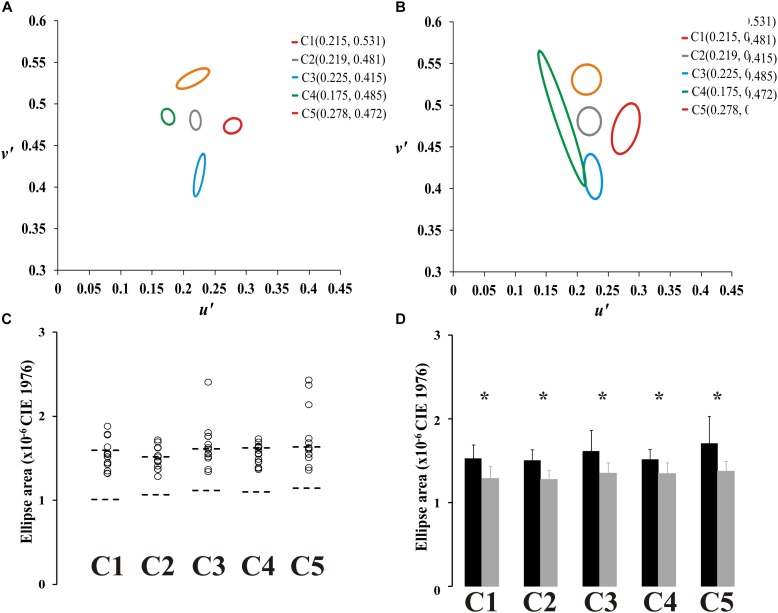
Results of Mollon-Reffin color discrimination test. **(A,B)** Color discrimination ellipses in the CIE1976 color space estimated from a control subject and an ex-alcoholic subject. **(C)** Scatter plots of the diameter of the circle with same area of the ellipse for five reference coordinates in the color space estimated from ex-alcoholic subjects. Dotted lines represent the tolerance interval of the control group. **(D)** Comparison of the mean diameter of the circle with same area of the ellipse for control group (gray bar) and ex-alcoholic group (black bar). Error bars represent the standard deviation. ^∗^*p* < 0.05.

Fourteen ex-alcoholics performed the Mollon–Reffin color discrimination test. Figure 3A,B shows the color discrimination ellipses in the CIE1976 color space estimated from a control subject (Figure 3A) and an ex-alcoholic subject (Figure 3B). Figure 3C presents the scatter plot of the ellipse area estimated from the ex-alcoholics for each reference chromaticity of the stimulus background. The data from ex-alcoholics are compared to the normative range of the control group for the same stimulus condition (dotted lines). For all central coordinates, the mean ellipse area of the ex-alcoholic group was significantly larger than the controls [Figure 3D, C1: *t*(45) = 5.07, *p* < 0.05; C2: *t*(45) = 5.79, *p* < 0.05; C3: *t*(45) = 3.89, *p* < 0.05; C4: *t*(45) = 4.49, *p* < 0.05; C5: *t*(45) = 4.05; *p* < 0.05].

### Association Between the Results of Different Psychophysical Tests

Table 1 shows a binary individual result from the ex-alcoholics we evaluated in the present study.

#### Association Between Contrast Sensitivity Function and Mollon–Reffin Color Discrimination Test

We found that two ex-alcoholics had normal color vision evaluated by MR test and 13 ex-alcoholic subjects had impaired color vision evaluated using MR test. We did not compare the contrast sensitivity between them due the low number of subjects in the group with normal color vision.

We grouped the results from MR test from ex-alcoholic subjects with normal (*n* = 9) or impaired (*n* = 5) contrast sensitivity at any spatial frequency. It resulted in no significant difference between the two groups at all reference coordinates (*p* > 0.05). However, when both groups were compared to the control group, the ex-alcoholic subjects with normal contrast sensitivity had larger ellipses than the controls for all reference coordinates (*p* < 0.05), while the ex-alcoholic subjects with impaired contrast sensitivity had larger ellipses for the reference coordinates C1, C2, C3, and C5 compared to the control group (*p* < 0.05).

#### Contrast Sensitivity Function and Farnsworth–Munsell 100 Hue Arrangement Test

**FIGURE 4:**
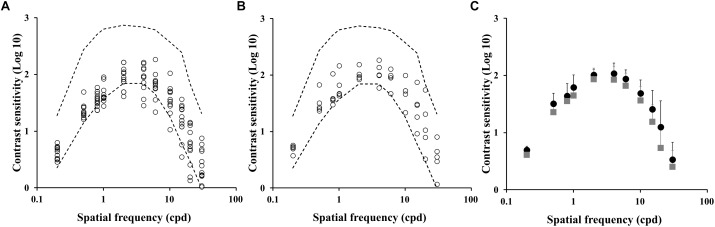
Spatial luminance contrast sensitivity functions from subjects with impaired and normal FM100 test results. **(A,B)** Scatter plots of the contrast sensitivity estimated from ex-alcoholic subjects with impaired and normal FM100 test results, respectively. Dotted lines represent the tolerance interval of the control group. **(C)** Comparison of the mean contrast sensitivity function of the subjects with impaired FM100 test result (gray squares) and normal FM100 test result (black circles). Error bars represent the standard deviation of the mean.

We compared the contrast sensitivity function from a group of ex-alcoholics with normal (*n* = 5) or impaired (*n* = 11) color vision evaluated by Farnsworth-Munsell 100 hue arrangement test (FM100). Figure 4A,B shows the contrast sensitivity values estimated from the impaired color vision group and normal color vision group, respectively. There was no difference between the contrast sensitivity estimated from both groups of alcoholic subjects (Figure 4C, *p* > 0.05). However, the comparison between the control group and ex-alcoholics with normal color vision estimated using FM100 showed contrast sensitivity impairment of the ex-alcoholics from 0.2 to 4 cpd, and 20 cpd (*p* < 0.05), while the same comparison between the controls and ex-alcoholics with impaired color vision evaluated by FM100 showed impaired contrast sensitivity at the ranges 0.2–4 cpd, and 15–30 cpd (*p* < 0.05).

**FIGURE 5:**
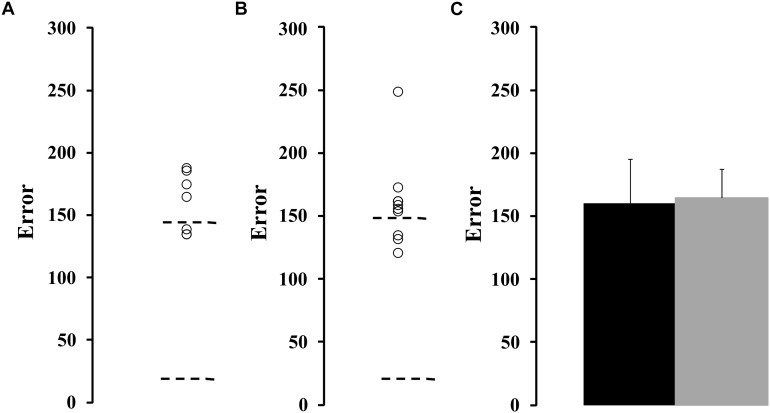
FM100 test results from subjects with normal and impaired contrast sensitivity spatial of luminance. **(A)** Results for subjects with impaired spatial luminance contrast sensitivity in, at least, one spatial frequency. **(B)** Results for subjects with normal contrast sensitivity. Dotted lines represent the tolerance interval of the control group. **(C)** Comparison of mean error value in subjects with impaired contrast sensitivity (gray squares) and normal contrast sensitivity (black circles). Error bars represent the standard deviation of the mean.

We also grouped the FM100 results from ex-alcoholic subjects with normal (*n* = 10) or impaired (*n* = 6) contrast sensitivity at any spatial frequency. No difference was found between both ex-alcoholic groups (Figure 5A–C, *p* > 0.05), but both groups had higher FM100 errors compared to the control group (*p* < 0.05).

#### Mollon–Reffin Color Discrimination Test and Farnsworth–Munsell 100 Hue Arrangement Test

**FIGURE 6:**
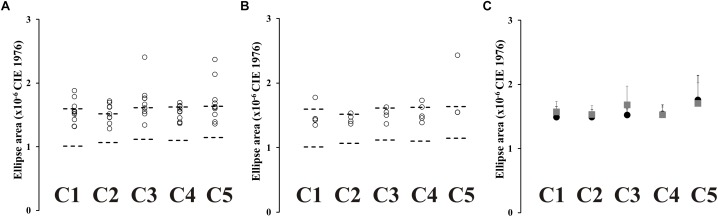
MR test results from subjects with normal and impaired FM100 test. **(A)** Scatter plots for the diameter of the circle with same area of the ellipse estimated from subjects with impaired FM100 results. **(B)** Scatter plots for the diameter of the circle with same area of the ellipse estimated from subjects with normal FM100 results. **(C)** Comparison of mean value of diameter of circle with same area of the ellipse in subjects with impaired FM100 test (gray squares) and normal FM100 test (black circles). Error bars represent the standard deviation of the mean.

We compared the color discrimination ellipse estimated from a group of ex-alcoholics with normal (*n* = 5) or impaired (*n* = 9) color vision evaluated by Farnsworth-Munsell 100 hue arrangement test. Figure 6A,B shows ellipse areas estimated from the impaired color vision group and normal color vision group evaluated by FM100 test, respectively. No difference was found between both alcoholic groups (Figure 6C, *p* > 0.05). The alcoholic group with normal FM100 results had larger ellipses than the controls for the central chromaticities C2 and C5, while the ex-alcoholic group with impaired FM100 results had larger ellipses than the controls for all central chromaticities (*p* < 0.05).

The mean data from FM100 test estimated from two ex-alcoholic subjects who had normal color vision evaluated by MR test and from 14 ex-alcoholic subjects with impaired results in the MR test. Here, we also did not perform statistical comparisons between the groups due the number of subjects in the group of normal results for MR test.

#### Multivariate Fisher Linear Discriminant Analysis

**FIGURE 7:**
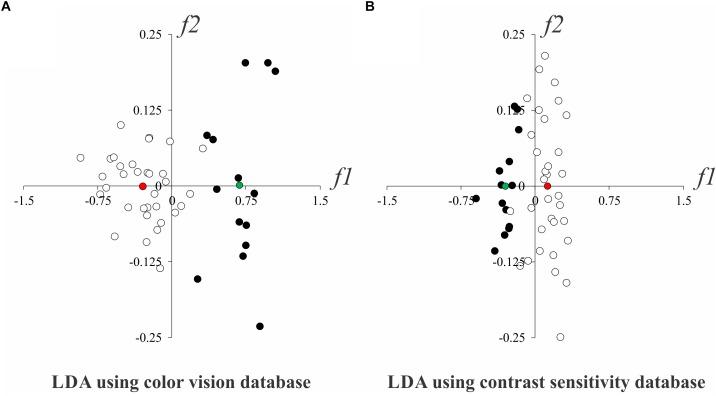
Linear separability of the control (white circles) and alcoholic (black circles) groups in LDA subspace. **(A)** LDA results using color vision evaluation results. **(B)** LDA results using contrast sensitivity evaluation results. Red and green circles represent the centroids from controls and alcoholic subjects, respectively. *f*1 and *f*2 represent the first two discriminant functions from LDA.

Table 2 shows in which visual test the participants had normal or altered results. As we found that contrast sensitivity and color vision evaluations were able to detect visual disturbances in alcoholics, we proceeded a multivariate linear discriminant analysis to identify which visual test could be better to separate the data from controls and alcoholics. Figure 7 shows the two-dimensional spaces of discriminants functions extracted from the analysis that used color vision (5 Mollon-Reffin test and FM100 score, Figure 7A) and contrast sensitivity results (11 spatial frequencies, Figure 7B), respectively. We observed that both results could separate the database from alcoholics and controls, and that the between-centroids distance from the analysis using color vision database was higher than that measured using the contrast sensitivity database (0.977 vs. 0.424, respectively).

**Table 2:** Individual results of the ex-alcoholics for each test.

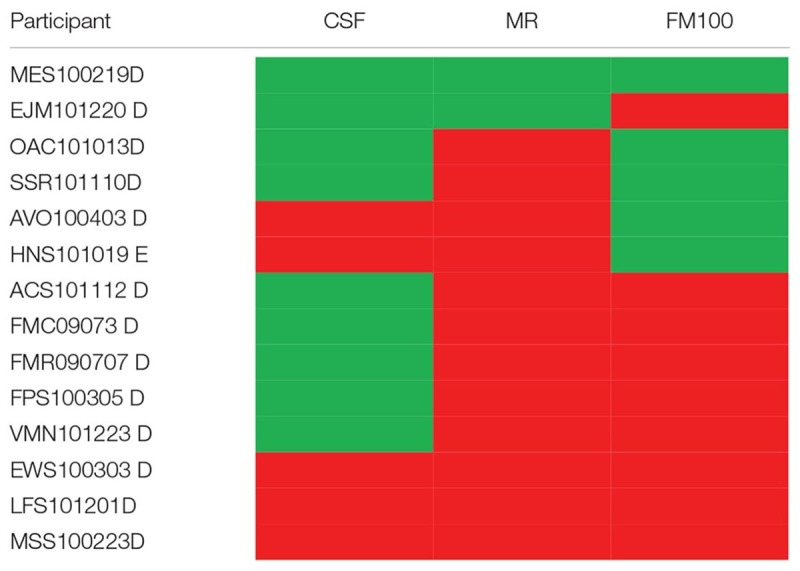

*Red cells indicate altered results; green cells indicate normal results. CSF, luminance contrast sensitivity function; MR, Mollon–Reffin test; FM 100, Farnsworth–Munsell test*.

## Discussion

Abusive alcohol consumption can cause physical, mental and social damage in humans (World Health Organization [WHO], 2000). In the current study, we have shown visual impairment in chronic alcoholic subjects during their abstinence period. These alterations were diagnosed even when they had no ophthalmologic clinic alterations. We also observed that most subjects present combined impairment of the color vision tests. The subjects presenting contrast sensitivity impairment also showed color vision impairment (in MR test). The subject who had only one impaired result had impairment in color vision test (EJM101220 D). A multivariate analysis showed that the color vision evaluation was better to separate the results from controls and alcoholics.

Color vision has been used to identify earlier effects of different chemicals on the visual system (Ventura et al., 2004; da Costa et al., 2008; Barboni et al., 2009; Feitosa-Santana et al., 2010; Lacerda et al., 2012; Brasil et al., 2015). Visual vulnerability to chemical exposure can be explained by the fact that while the fovea is the main region of the human retina to process color information (Roorda and Williams, 1999; Brainard et al., 2000), this area has limited vascularization in order to provide good optical media to the light reaching on the retina. In conditions of cellular or metabolic aggression, the foveal region becomes more susceptible to oxidative stress than the retinal periphery (Izzotti et al., 2006; Izuta et al., 2010). In addition, the number of color opponent cells (red-green and blue-yellow opponency) is lower than the number of cells that process luminance opponency along the visual pathways (Brainard et al., 2000). We do not discard the explaining hypothesis that in primary visual cortex the color information is processed in regions named “blobs,” that have a high activity of cytochrome oxidase, an enzyme involved in the cellular mechanisms of energy production (Boyd and Casagrande, 1999). Experimental studies which evaluated the effect of alcohol on the visual system of rat described an increase in optic nerve oxidative stress (Aviñó et al., 2002). In monkeys (*Macaca mulatta*) ethanol metabolism in neural tissue and retina was found to change tissue fatty acid and to increase lipid oxidation, contributing to oxidative stress associated with retinal function impairment (Pawlosky et al., 2001).

Initially, the studies that investigated involvement of color vision in the neural damage caused by alcoholism showed selective losses in blue-yellow mechanisms (Cruz-Coke and Varela, 1965; Thuline, 1967; Varela et al., 1969; Sassoon et al., 1970; Cruz-Coke, 1972; Adams et al., 1975; Russell et al., 1980; Verriest et al., 1980; Zrenner et al., 1986). Some other investigations reported red-green or diffuse losses (Sarraux et al., 1966; Smith and Layden, 1971; Rothstein et al., 1973; Sakuma, 1973; Mergler et al., 1988; Kapitany et al., 1993; Castro et al., 2009). We found no preference for any chromatic mechanisms even using two kinds of color tests. FM100 is a task with suprathreshold stimulus, while MR estimates threshold measurements of the color vision. For both tests the visual losses were similar across the chromatic axes.

It has been well-established that changes in neurocognitive networks and concomitant deficits in cognitive function are common in former alcoholics (Pandey et al., 2018). It is reasonable to think that both evaluations of color processing would seem to have a higher cognitive demand than the evaluation of spatial contrast sensitivity, since they require more complex instructions and judgements (i.e., choose the hue closest to the reference hue, which of four orientations is the gap in the C facing, versus “do you see the stimulus or not”). We did not evaluate cognitive functions of our ex-alcoholic sample, but we consider that our results represent the consequence of sensory impairment. Considering the example of the Mollon-Reffin test, the ex-alcoholics could perform the behavioral task in stimulus condition with highly saturated chromaticities and failed in less saturated chromaticity condition (but above the normative thresholds). So, we concluded that they understood the task and their main limitation was perceptual. The same rationale we can extrapolate to the hue ordering test.

A limitation of the present study is the sample size of the ex-alcoholic group, although with this sample size we could find significant differences compared to the controls. Other thing that is important to emphasize is that this sample has no other important clinical complication associated to the abuse of alcohol consume in the past.

The literature diverges as to the possibility of abstinence from alcohol being able to reduce the damage caused by the chronic alcoholism in specific functions of the central nervous system. There is the suggestion that after a prolonged time of abstinence from alcohol, the brain may reorganize to compensate behavioral and structural deficits (Oscar-Berman et al., 2014) including increase in cortical thickness in the brain’s extended reward and oversight system (Durazzo et al., 2011), recovery in brain volume, microstructure, and neurochemistry (Oscar-Berman et al., 2014) improvement of gait, balance and neuropsychological functions (Fein et al., 2006), but some deficits have been found to persist, such as problems with executive functioning, motor functions and visuospatial cognition (Smith and Fein, 2011; Oscar-Berman et al., 2014). The color vision functions might be one of these persistent alterations induced by chronic alcoholism even after long abstinence, such as found in the present study.

## Author Contributions

All authors contributed to the conception of the work, and in drafting and revising the manuscript. All authors have approved the final version and agree to be accountable for all aspects of the work. AOC and LCLS designed the experiments. The psychophysical experiments were carried out by ICVSM, AB, EMCBL, and ARR. The data were analyzed by GSS, AB, and ICVSM. Components of the manuscript were written by GSS, DV, and AH.

## Conflict of Interest Statement

The authors declare that the research was conducted in the absence of any commercial or financial relationships that could be construed as a potential conflict of interest.
